# Salinity Modulates Carbon Flux to Promote Squalene and PUFA Biosynthesis in the Marine Protist *Thraustochytrium*

**DOI:** 10.3390/md23090354

**Published:** 2025-08-30

**Authors:** Yuetong Zhao, Xingyu Zhu, Nimra Riaz, Xiuping Liu, Jiaqian Li, Guangyi Wang

**Affiliations:** 1 Center of Marine Environmental Ecology, School of Environmental Science and Engineering, Tianjin University, Tianjin 300072, China; 2023214029@tju.edu.cn (Y.Z.); 1021214036@tju.edu.cn (X.Z.); nimrariaz.505@gmail.com (N.R.); liuxp880@tju.edu.cn (X.L.); lijiaqian@tju.edu.cn (J.L.); 2 Key Laboratory of Systems Bioengineering (Ministry of Education), Tianjin University, Tianjin 300072, China

**Keywords:** salinity stress, thraustochytrids, squalene and fatty acid biosynthesis, energy metabolism

## Abstract

Salinity is a key environmental factor regulating lipid metabolism in marine oleaginous protists. This study examined the impact of NaCl concentration on growth, glucose utilization, and lipid biosynthesis in *Thraustochytrium* sp. ATCC 26185. Moderate salinity (20 g/L) enhanced biomass and glucose uptake, while high salinity (45 g/L) induced osmotic stress yet significantly promoted squalene accumulation (17.27 mg/g), a 3.26-fold increase compared with 0 g/L NaCl (5.29 mg/g). Integrated transcriptomic and metabolomic analyses revealed that salinity-dependent activation of glycolysis, the TCA cycle, and the pentose phosphate pathway increased cellular ATP, NADH, and NADPH levels. Under salt stress, the mevalonate (MVA) pathway was transcriptionally upregulated, with key enzymes, including ACAT, HMGR, and IDI, showing marked induction, which supports enhanced carbon flux toward squalene biosynthesis. Despite SQS downregulation, squalene accumulation increased, likely due to elevated precursor availability and reduced flux to downstream sterol pathways. Concurrently, high salinity repressed expression of ACC, FAS-α, and FAS-β, reducing saturated fatty acid levels, while upregulation of PKSB-favored polyunsaturated fatty acid (PUFA) synthesis. These findings suggest that high-salt stress triggers transcriptional reprogramming, redirecting acetyl-CoA from fatty acid synthesis toward squalene and PUFA production. This study offers new insights into the metabolic plasticity of thraustochytrids and highlights salinity modulation as a promising strategy for enhancing high-value lipid yields in marine biotechnology.

## 1. Introduction

Thraustochytrids are unicellular, heterotrophic marine protists that play essential roles in oceanic carbon cycling and microbial food webs [1,2]. These microorganisms inhabit diverse saline environments, from coastal estuaries to open oceans, where salinity critically shapes their distribution, growth, and ecological functions [3,4]. Although thraustochytrids are generally adaptable, optimal NaCl concentrations for biomass production are strain-specific. For example, *Schizochytrium limacinum* OUC88 grows best at 28 g/L NaCl, while *Thraustochytrium* sp. ATCC 26185 and *Aurantiochytrium* sp. TWZ-97 perform optimally at 5 g/L and 7.5 g/L, respectively [5,6,7]. Excessive salinity can cause osmotic stress, compromise membrane integrity, and halt cell growth, as observed in *Thraustochytrium striatum* ATCC 24473 under double seawater salinity [8]. Notably, some strains tolerate near-freshwater conditions, and recent phylogenetic studies have reclassified several low-salinity-adapted genera, such as *Diplophrys* and *Paramphitrema*, into the family *Amphitraemidae* [5,9,10,11]. Coastal salinity gradients further shape thraustochytrid diversity and ecological specialization. Despite growing interest, the full extent of salinity’s impact on thraustochytrid physiology and metabolism remains insufficiently understood.

Beyond ecological adaptation, NaCl plays a crucial role in regulating thraustochytrid metabolism. Sodium ions (Na^+^) contribute to ionic homeostasis, modulate enzymatic activity, and influence energy production [12,13]. At moderate salinity (5 g/L NaCl), Na^+^ enhances ATP generation through fatty acid β-oxidation and promotes squalene biosynthesis via the mevalonate pathway [14]. Transcriptomic analyses reveal upregulation of the salinity-responsive genes involved in lipid metabolism, highlighting the metabolic plasticity of thraustochytrids under changing salt conditions [15]. While similar stress responses have been documented in marine microbes such as *Prochlorococcus*, the molecular mechanisms driving Na^+^-mediated metabolic shifts and high-salinity tolerance in thraustochytrids remain underexplored [16].

Thraustochytrids are known for synthesizing a range of high-value metabolites, including the triterpenoid squalene, polyunsaturated fatty acids (PUFAs), and carotenoids [17]. Among these, squalene is a natural triterpenoid compound with diverse biological functions, such as antioxidation, anti-tumor activity, and immune enhancement [18]. It has broad applications in pharmaceuticals, cosmetics, and health supplements [19,20]. In addition, the PUFAs produced by thraustochytrids are primarily docosahexaenoic acid (DHA) and eicosapentaenoic acid (EPA), two ω-3 fatty acids that play critical roles in neural development and cardiovascular health [21]. Growing demand for these high-value lipids, coupled with the limitations of plant and animal sources, has accelerated the transition toward microbial production. Thraustochytrids, in particular, have emerged as promising cell factories, offering inherent biosynthetic capacity, rapid growth, independence from arable land and seasonal cycles, and suitability for continuous cultivation in bioreactors. Optimizing their cultivation conditions is therefore critical for realizing economically viable and sustainable microbial cell factories.

NaCl concentration also profoundly influences the biosynthesis of commercially valuable metabolites, including polyunsaturated fatty acids and the triterpenoid squalene, by modulating carbon flux between these lipid classes. For PUFAs like docosahexaenoic acid (DHA), low salinity often reduces its production. For instance, at 1 mM NaCl, *Thraustochytrium* strain ACEM A shifts its fatty acid profile toward saturated fatty acids (C14:0, C16:0), significantly decreasing PUFA levels [11,17]. Conversely, intermediate to high salinities favor squalene accumulation. *Aurantiochytrium* sp. TWZ-97 produces 456.3 mg/L squalene at 7.5 g/L NaCl—over twice the yield obtained at lower salinity [5]. Similarly, *Aurantiochytrium* sp. 18W-13a achieves maximum squalene production at 25–50% seawater salinity [18]. These findings highlight NaCl as a critical factor for optimizing the metabolic output of thraustochytrids in the industrial production of nutraceuticals such as ω-3 fatty acids and terpenoids. However, high salinity accelerates the corrosion of fermentation equipment [19,20]. Thus, it is of vital importance to control salinity in large-scale cultivation.

In this study, we systematically investigated the effects of NaCl concentration on the growth, glucose consumption, fatty acid composition, and squalene production of *Thraustochytrium* sp. ATCC 26185. By integrating transcriptomic and metabolomic analyses, we characterized the molecular responses to high-salinity stress and elucidated the mechanisms linking elevated NaCl levels to enhanced squalene biosynthesis. This work addresses an important knowledge gap and provides the first comprehensive insights into how high-salinity environments regulate thraustochytrid physiology and metabolism, advancing their potential for industrial lipid production.

## 2. Results and Discussion

### 2.1. Effects of NaCl on Growth and Glucose Utilization

**Figure 1:**
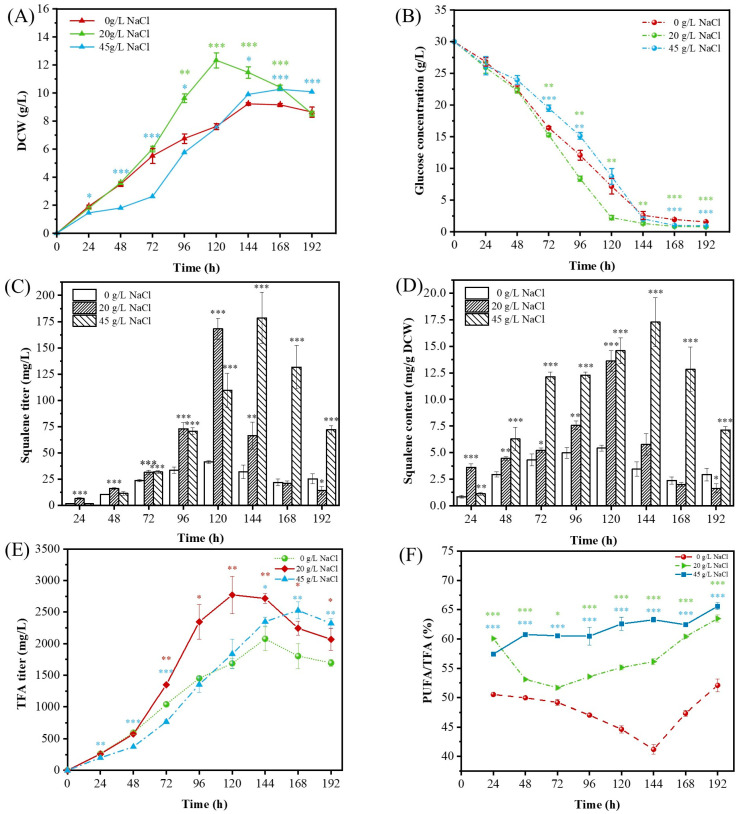
Effects of different NaCl concentrations on (**A**) dry cell weight (DCW), (**B**) glucose concentration, (**C**) squalene titer, (**D**) squalene content, (**E**) total fatty acid (TFA) concentration, and (**F**) polyunsaturated fatty acid (PUFA) content in *Thraustochytrium* sp. ATCC 26185. Each bar represents the mean ± SD of three independent experiments. * indicates *p* < 0.05, ** indicates *p* < 0.01, and *** indicates *p* < 0.001 (relative to the control group).

Salinity is a well-established environmental factor shaping the physiology and metabolite production of thraustochytrids [21]. To examine the effects of NaCl concentration on the growth dynamics of *Thraustochytrium* sp. ATCC 26185, cultures were cultivated under three salinity levels (0, 20, and 45 g/L NaCl), designated as low, moderate, and high salinity, respectively, relative to the basal medium containing 25 g/L NaCl. Biomass and residual glucose were monitored every 24 h. The strain exhibited robust growth across all treatments, but with distinct growth kinetics (Figure 1A). Exponential growth persisted until 120 h in all groups. However, cultures grown at 20 g/L NaCl entered the stationary phase earlier than those at 0 and 45 g/L NaCl, which continued growing up to 144 h. The highest biomass yield (12.34 g/L) was achieved at 20 g/L NaCl, while the lowest (9.22 g/L) occurred at 0 g/L, indicating that moderate salinity optimally supports biomass accumulation. These results are consistent with previous reports showing that thraustochytrids grow best in media simulating half-strength seawater [22,23].

Earlier studies similarly observed that low-salinity conditions (1–10 mM NaCl) could reduce biomass by 15–35% in *Japonochytrium* sp. ATCC 28207, a close relative of *Schizochytrium limacinum*, though growth could be partially restored by supplementing compatible solutes such as mannitol and sucrose [11,24]. Moreover, ion-selective microelectrode studies have demonstrated that Na^+^ accounts for over half of the osmotic adjustment in marine protists like strain ACEM C, with chloride ions serving as secondary osmolytes [24]. These findings emphasize NaCl’s dual role in osmoregulation and metabolic homeostasis. However, its broader physiological and molecular effects on growth and substrate utilization in thraustochytrids like *Thraustochytrium* sp. ATCC 26185 have remained poorly characterized.

Given that glucose uptake is a primary determinant of cell metabolism and growth, residual glucose concentrations were tracked to assess how salinity influences carbon utilization. While all treatments displayed similar overall consumption patterns, significant differences emerged during the exponential growth phase. The fastest glucose consumption was observed at 20 g/L NaCl, while the slowest occurred at 45 g/L (Figure 1B). By the time cultures reached the stationary phase, over 91% of glucose was depleted in all groups. However, at 120 h, the cultures at 20 g/L NaCl had already consumed >90% of the available glucose, while 20–30% remained in the 0 and 45 g/L treatments. These results indicate that moderate salinity enhances glucose uptake efficiency, likely contributing to higher biomass accumulation under these conditions.

Similar trends have been reported when glucose utilization was stimulated by exogenous antioxidant additives, such as melatonin, mannitol, and ascorbic acid, in both *Thraustochytrium* sp. ATCC 26185 and *Schizochytrium* sp. HX-308 [25,26]. Notably, this is the first study to directly demonstrate that NaCl concentration alone significantly influences glucose uptake rates in *Thraustochytrium* sp. ATCC 26185. The strong correlation between salinity-dependent differences in growth and glucose utilization suggests that moderate NaCl concentrations optimize cellular metabolism and resource assimilation, thereby promoting biomass productivity.

Clearly, the NaCl concentration profoundly influences both growth and glucose utilization in *Thraustochytrium* sp. ATCC 26185. Optimal biomass production and the fastest glucose uptake occurred at 20 g/L NaCl, while higher (45 g/L) and lower (0 g/L) salinities resulted in slower growth and delayed glucose consumption. It is worth noting that, as a marine protist, *Thraustochytrium* sp. ATCC 26185 is inherently adapted to saline environments. Low-salt conditions (0 g/L NaCl) may disrupt ion homeostasis and membrane integrity, thereby inducing osmotic stress comparable to or even greater than that under high salinity. Previous studies have shown that low salinity can lead to reduced growth and altered lipid profiles in thraustochytrids, likely due to compromised osmoregulation. In the present study, although not explicitly measured, the observed reduction in biomass and delayed glucose uptake at 0 g/L NaCl may reflect such low-salt stress. These findings suggest that moderate salinity optimizes osmotic balance and cellular metabolism, promoting efficient carbon assimilation and biomass accumulation. This salinity-dependent regulation of substrate uptake mirrors observations in other thraustochytrids and marine protists and underscores the importance of fine-tuning salinity to enhance both growth performance and metabolic activity in thraustochytrid cultivation systems [22,24].

### 2.2. Effects of NaCl Concentration on Squalene and Fatty Acid Production

Beyond regulating growth, salinity is a critical factor influencing lipid metabolism and squalene biosynthesis in thraustochytrids [6,18]. To evaluate the effect of NaCl concentration on these processes, *Thraustochytrium* sp. ATCC 26185 was cultivated under three salinity conditions (0, 20, and 45 g/L NaCl), with squalene production, total fatty acid (TFA) production, and PUFA content monitored over time (Figure 1C–F).

Squalene accumulation increased progressively during cultivation in all treatments, peaking during the stationary phase before declining—a pattern consistent with previous observations in *Aurantiochytrium* sp. T66 and *Schizochytrium* sp. HX-308 [27,28]. Peak squalene titers at 0 and 20 g/L NaCl were reached at 120 h, whereas at 45 g/L NaCl, maximum levels appeared at 144 h (Figure 1C). Notably, peak titers under 20 g/L (168.01 mg/L) and 45 g/L (178.45 mg/L) NaCl were approximately fourfold higher than those at 0 g/L (41.22 mg/L), indicating a clear salinity-dependent enhancement of squalene biosynthesis. Although biomass production was slightly reduced at 45 g/L NaCl, squalene accumulation was highest under this condition, suggesting that elevated NaCl promotes squalene biosynthesis independently of biomass. Interestingly, at 45 g/L NaCl, squalene content rose more rapidly during the early cultivation phase (24–72 h), coinciding with the strain’s adaptation to hyperosmotic stress (Figure 1D). This early surge suggests that squalene, as a key precursor to the sterols essential for membrane integrity, may contribute to osmotic stress adaptation. Alternatively, squalene may itself function as a lipophilic antioxidant, protecting the cell from oxidative damage associated with hyperosmotic stress, as has been proposed in other biological systems [29,30].

NaCl concentration also influenced TFA production (Figure 1E). Maximum TFA yields under 0, 20, and 45 g/L NaCl were 2.08, 2.77, and 2.53 g/L, respectively. Moderate salinity (20 g/L NaCl) was optimal for TFA accumulation, producing consistently higher yields between 72 h and 144 h. In all treatments, TFA content increased with cultivation time but exhibited non-linear trends in response to salinity. While the absence of NaCl initially supported relatively high TFA yields, levels declined after 96 h. Conversely, at 45 g/L NaCl, TFA synthesis was initially suppressed, likely due to osmotic stress, but recovered after 72 h as cells adapted, mirroring biomass trends. Similar salt-responsive lipid accumulation patterns have been reported in other thraustochytrids. In *Thraustochytrium striatum* ATCC 24473, TFA content increased from 4% to 11% as artificial seawater salinity rose from 25% to 100% [8]. Likewise, *Schizochytrium mangrovei* Sk-02 showed a rise in TFA content from 51.2% to 57.3% when the sea salt concentration increased from 0 to 30 g/L [31].

The PUFA content also exhibited a salinity-dependent variation (Figure 1F). Under 0 g/L NaCl, PUFA levels remained below 50% throughout cultivation. In contrast, at 20 g/L and 45 g/L NaCl, PUFA content consistently exceeded 50%, surpassing 60% at later time points. At both 0 and 20 g/L NaCl, PUFA levels followed a “V-shaped” trend—declining initially and recovering later. Specifically, PUFA content under 0 g/L NaCl decreased from 50.52% at 24 h to 41.19% at 144 h, then rebounded to 52.09% at 192 h. This pattern of PUFA content under 0 g/L NaCl may reflect a shift in lipid remodeling: early dominance of SFA synthesis for structural integrity, followed by increased PUFA production to enhance membrane fluidity and oxidative defense under prolonged low-salt stress. At 20 g/L, it dropped from 60.08% at 24 h to 51.68% at 72 h, then gradually increased to 63.49% at 192 h. In contrast, under 45 g/L NaCl, PUFA content steadily increased throughout cultivation, from 57.42% at 24 h to 65.60% at 192 h. These results align with earlier findings in *Spirulina* strains, where PUFA accumulation was enhanced by elevated salinity [32]. Given that fatty acid composition affects membrane fluidity, increased PUFA levels under high salinity likely contribute to osmotic stress adaptation while also supporting energy storage and antioxidant capacity [33,34].

Overall, NaCl concentration exerts a profound influence on both squalene and total fatty acid biosynthesis in *Thraustochytrium* sp. ATCC 26185. While moderate salinity (20 g/L NaCl) favored overall biomass and TFA production, higher salinity (45 g/L) significantly enhanced squalene and PUFA accumulation, likely as part of a broader stress-adaptive response. These findings highlight the dual regulatory role of salinity in modulating metabolite synthesis and cellular stress resilience. Importantly, they underscore the potential of salinity-based bioprocess optimization for boosting high-value lipid yields in marine protist cultivation systems.

### 2.3. Transcriptomic and Metabolomic Insights into NaCl-Regulated Squalene and Lipid Biosynthesis

#### 2.3.1. Sequencing Data Quality Assessment

Consistent with previous studies, our results demonstrated that squalene and total fatty acid (TFA) accumulations in *Thraustochytrium* sp. ATCC 26185 are closely associated with NaCl concentration, with enhanced production observed under high-salinity conditions. To investigate the underlying molecular mechanisms, cultures grown at 0, 20, and 45 g/L NaCl were sampled at 72 h and 144 h for transcriptomic analysis. The samples were divided into six groups: LS72 (72 h, 0 g/L NaCl), NS72 (72 h, 20 g/L NaCl), HS72 (72 h, 45 g/L NaCl), LS144 (144 h, 0 g/L NaCl), NS144 (144 h, 20 g/L NaCl), and HS144 (144 h, 45 g/L NaCl). These time points were strategically chosen to capture distinct physiological and metabolic states. At 72 h, cells were in the logarithmic growth phase, characterized by active proliferation and peak metabolic activity, thereby reflecting the dynamic impact of salinity on basal metabolism. At 144 h, cells had reached the stationary phase, with high biomass and maximal metabolite accumulation, providing insights into how salinity stress influences carbon flux allocation toward high-value lipid synthesis pathways. Following RNA extraction, sequencing, and quality filtering, each group yielded between 38.5 and 41.2 million clean reads, with the Q30 values exceeding 93%, indicating high sequencing accuracy and reliability (Table 1). The GC content ranged from 63.61% to 63.92%. De novo assembly generated 27,812 unigenes totaling 33.1 Mbp, with an N50 of 2110 bp and a GC content of 62.65%, confirming the quality of the assembly for downstream analysis (Table 2).

Comparative transcriptomic analysis revealed substantial changes in gene expression across salinity treatments. Specifically, 2649 (NS72_vs_LS72), 2935 (HS72_vs_LS72), 1312 (HS72_vs_NS72), 4012 (NS144_vs_LS144), 4542 (HS144_vs_LS144), and 1407 (HS144_vs_NS144) differentially expressed genes (DEGs) were identified in the respective pairwise comparisons (Table 3). We focused on salt vs. no-salt contrasts (LS as control) to elucidate NaCl-specific effects, while NS vs. HS comparisons were omitted from the pathway-level analyses due to consistently lower DEG counts between 20 and 45 g/L NaCl (1312 at 72 h and 1407 at 144 h). This pattern suggests that moderate and high salinity elicit more similar transcriptional responses compared to no salinity, highlighting NaCl’s broad regulatory role in cellular gene expression under both low- and high-salt conditions.

To complement transcriptomic findings, metabolomic analyses were conducted on the same samples using positive and negative ion modes. A total of 7907 metabolic ions were detected in negative mode, with 4321 annotated to known metabolites, including 3640 matched to the HMDB database and 2921 to the KEGG database (Table 4). In positive mode, 14,410 ions were identified, with 7635 annotated metabolites, 6659 HMDB entries, and 4887 KEGG annotations. Differential metabolite analysis revealed 4711 (NS72_vs_LS72), 5238 (HS72_vs_LS72), 1491 (HS72_vs_NS72), 5458 (NS144_vs_LS144), 5919 (HS144_vs_LS144), and 1020 (HS144_vs_NS144) significantly altered metabolized ions across the respective comparisons. Similar to the transcriptomic results, the number of differentially metabolized ions between 20 and 45 g/L NaCl was markedly lower than that between each of these treatments and 0 g/L NaCl. This further supports the conclusion that moderate and high salinity have comparable effects on cellular metabolism, distinct from the response under no-salt conditions.

Additionally, 403 negative ions and 716 positive ions were annotated as secondary metabolites, providing further evidence of the salinity-induced modulation of key biosynthetic pathways, including those involved in lipid and squalene production (Table 4). Together, these integrated omics results offer new insights into how NaCl regulates gene expression and metabolism in *Thraustochytrium* sp. ATCC 26185, contributing to enhanced lipid biosynthesis under saline stress.

#### 2.3.2. High-Salt Stress Enhances Cellular Energy Metabolism via Coordinated Activation of Central Carbon Pathways and Oxidative Phosphorylation

**Figure 2:**
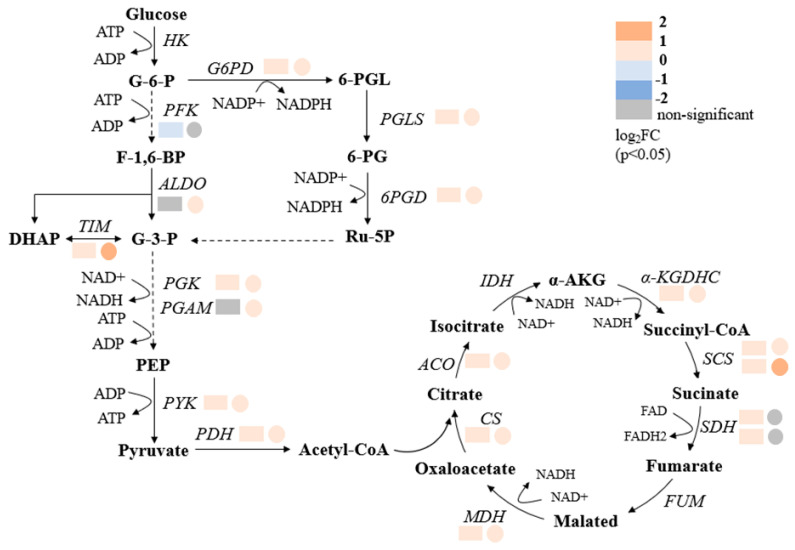
Transcriptomic analysis of the central carbon metabolism (CCM) pathway in *Thraustochytrium* sp. ATCC 26185 under two comparison groups: NS72 vs. LS72 and HS72 vs. LS72. In each case, the rectangle on the left represents changes in gene expression in the NS72 vs. LS72 group, while the circle on the right indicates changes in the HS72 vs. LS72 group. Abbreviations: G-6-P, glucose-6-phosphate; F-1,6-BP, fructose-1,6-bisphosphate; G-3-P, glyceraldehyde-3-phosphate; PEP, phosphoenolpyruvate; HK, hexokinase; PFK, phosphofructokinase; ALDO, fructose-bisphosphate aldolase; TIM, triose-phosphate isomerase; PGK, phosphoglycerate kinase; PGAM, phosphoglycerate mutase; PYK, pyruvate kinase; PDH, pyruvate dehydrogenase; CS, citrate synthase; ACO, aconitate hydratase; IDH, isocitrate dehydrogenase; α-KGDHC, α-ketoglutarate dehydrogenase complex; SCS, succinyl-CoA synthetase; SDH, succinate dehydrogenase; FUM, fumarase; MDH, malate dehydrogenase; G6PD, glucose-6-phosphate dehydrogenase; PGLS, 6-phosphogluconolactonase; and 6PGD, 6-phosphogluconate dehydrogenase.

**Figure 3:**
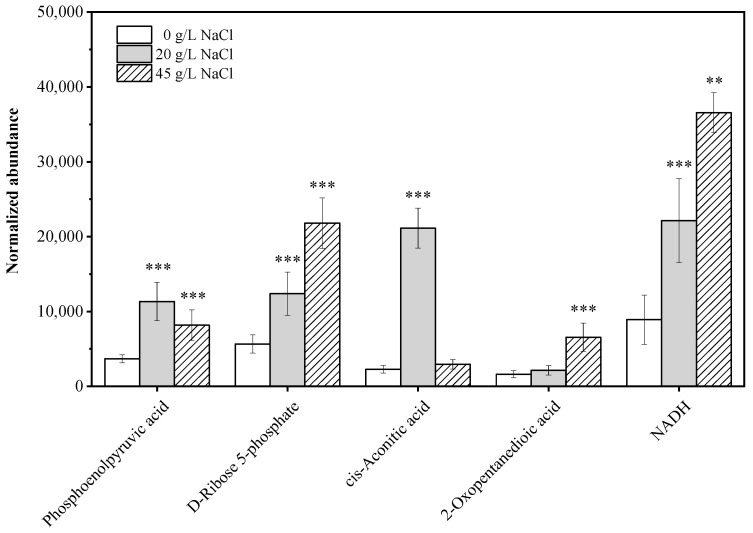
Contents of intermediate metabolites in the central carbon metabolism (CCM) pathway of *Thraustochytrium* sp. ATCC 26185 under different NaCl concentrations. Each bar represents the mean ± SD of three independent experiments. ** indicates *p* < 0.01, and *** indicates *p* < 0.001 (relative to the control group).

**Figure 4:**
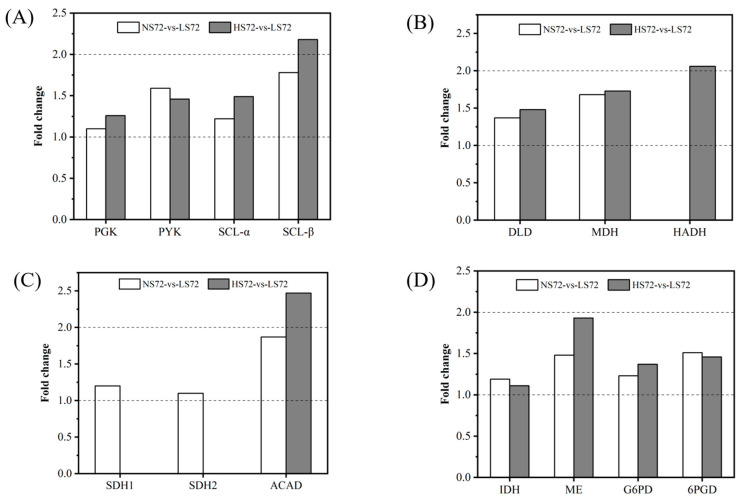
Gene expression profiles related to ATP and NADPH production in the central carbon metabolism (CCM) pathway of *Thraustochytrium* sp. ATCC 26185 across different comparison groups: (**A**) Genes involved in ATP and GTP synthesis; (**B**) genes related to NADH generation; (**C**) genes contributing to FADH_2_ production; and (**D**) genes associated with NADPH production. Abbreviations: PGK, phosphoglycerate kinase; PYK, pyruvate kinase; SCL, succinyl-CoA synthetase; IDH, isocitrate dehydrogenase; ME, malic enzyme; G6PD, glucose-6-phosphate dehydrogenase; 6PGD, 6-phosphogluconate dehydrogenase; DLD, dihydrolipoamide dehydrogenase; MDH, malate dehydrogenase; HADH, hydroxyacyl-CoA dehydrogenase; SDH, succinate dehydrogenase; ACAD, acyl-CoA dehydrogenase.

High-salt environments impose significant osmotic and oxidative stress on microbial cells, increasing the demand for energy to maintain homeostasis, osmotic balance, and biosynthetic activity [32]. To meet these heightened energy requirements, organisms often reprogram central metabolic pathways, including central carbon metabolism (CCM) and oxidative phosphorylation, which serve as the primary sources of ATP, NADH, and NADPH [35,36]. In this study, the transcriptomic and metabolomic analyses consistently demonstrated that NaCl concentrations of 20 and 45 g/L induced extensive upregulation of energy metabolism-related genes and metabolites in *Thraustochytrium* sp. ATCC 26185 (Figure 2, Figure 3 and Figure 4).

Transcriptome profiling revealed that genes involved in glycolysis, the tricarboxylic acid (TCA) cycle, and the pentose phosphate pathway (PPP) were upregulated under both 20 and 45 g/L NaCl (Figure 2). Notably, key enzymes such as isocitrate dehydrogenase (IDH), a crucial NAD(P)H-producing enzyme in the TCA cycle, increased by 1.19 and 1.11 fold, while 6-phosphogluconate dehydrogenase (6PGD), a major NADPH generator in the PPP, rose by 1.51 and 1.46 fold, respectively (Figure 2). These changes suggest that high-salt stress stimulates flux through multiple branches of central carbon metabolism, enhancing the supply of energy and reducing the equivalents essential for cellular adaptation.

Supporting this, metabolomic data indicated that intermediates of the CCM pathway, including phosphoenolpyruvate and D-ribose 5-phosphate, significantly accumulated under high-salt conditions. Phosphoenolpyruvate, a glycolytic intermediate involved in ATP generation and precursor supply for various biosynthetic pathways, increased by 3.08 and 2.22 fold at 20 and 45 g/L NaCl, respectively, while D-ribose 5-phosphate, a critical PPP metabolite, rose by 2.19 and 3.86 fold (Figure 3). This accumulation reflects enhanced metabolic fluxes through both glycolysis and the PPP, in line with the observed gene expression patterns.

Energy generation via substrate-level phosphorylation also appeared to be upregulated, as the genes encoding phosphoglycerate kinase (PGK), pyruvate kinase (PYK), and succinyl-CoA synthetase (SCS) displayed increased expression under high-salt conditions (Figure 4A). This would directly contribute to elevated ATP synthesis, crucial for maintaining ion gradients, osmolyte transport, and stress-responsive biosynthetic processes.

Oxidative phosphorylation, responsible for producing most of the cellular ATP, was likewise activated in response to NaCl stress. The genes encoding NADH-producing enzymes—including IDH, dihydrolipoamide dehydrogenase (DLD), and malate dehydrogenase (MDH) in the TCA cycle, along with 3-hydroxyacyl-CoA dehydrogenase (HADH) in fatty acid β-oxidation—were significantly upregulated (Figure 4B). Concurrently, the genes encoding FADH_2_-producing enzymes, such as succinate dehydrogenase (SDH) and acyl-CoA dehydrogenase (ACAD), were also induced (Figure 4C). Correspondingly, NADH levels increased by 2.49 and 4.11 fold at 20 and 45 g/L NaCl, respectively (Figure 3), confirming that the oxidative phosphorylation activity was enhanced to meet the greater energetic demands imposed by high salinity.

In addition to ATP and NADH, NADPH is a critical cofactor for anabolic reactions and antioxidant defense, particularly under osmotic and oxidative stress [37]. Here, the expression of the genes encoding NADPH-generating enzymes—including cytoplasmic IDH, malic enzyme (ME), glucose-6-phosphate dehydrogenase (G6PD), and 6PGD—was elevated under high-salt conditions (Figure 4D), with increases ranging from 1.00 to 2.00 fold. The overexpression of G6PD and 6PGD under salinity and low-temperature stress has been previously reported as a key mechanism to maintain intracellular NADPH homeostasis and antioxidant capacity, consistent with our findings [37,38].

In summary, our integrated transcriptomic and metabolomic analyses reveal that *Thraustochytrium* sp. ATCC 26185 responds to high-salinity stress by coordinately enhancing central carbon metabolism, substrate-level phosphorylation, and oxidative phosphorylation to meet elevated energy and redox demands. Upregulation of glycolysis, the TCA cycle, and the pentose phosphate pathway facilitated increased production of ATP, NADH, FADH_2_, and NADPH, supporting biosynthesis and stress tolerance under osmotic challenge. While previous studies on thraustochytrids have employed transcriptomics to explore salt stress responses or metabolomics to profile lipid accumulation, few have combined both omics layers to elucidate mechanism-level changes. Transcriptomics was used to identify salt-responsive genes in *Schizochytrium* sp., but did not correlate these with metabolic fluxes or energy metabolism [15]. Our study is among the first to integrate transcriptome and metabolome data to directly link NaCl-induced activation of central carbon metabolism, ATP/NADPH production, and transcriptional reprogramming of the MVA and PKS pathways in a thraustochytrid. This metabolic adjustment mirrors adaptive strategies reported in other marine microbes and halotolerant organisms, where salinity-induced activation of energy metabolism ensures survival in fluctuating environments [32,37,38,39]. Thus, high-salt exposure triggers an integrated reprogramming of energy metabolism, enabling thraustochytrids to maintain redox balance and bioenergetic stability in hypersaline environments. Also, these findings highlight the metabolic plasticity of thraustochytrids and provide valuable insights into their resilience in hypersaline marine environments.

#### 2.3.3. High-Salt Stress Regulates Squalene and Fatty Acid Biosynthesis

Squalene accumulation in *Thraustochytrium* sp. ATCC 26185 was positively correlated with NaCl concentration, reaching 5.29, 13.60, and 17.27 mg/g at 0, 20, and 45 g/L NaCl, respectively, at 144 h (Figure 1C). To elucidate the molecular basis of this trend, transcriptome analysis was conducted to evaluate the expression of genes in the mevalonate (MVA) pathway, which governs squalene biosynthesis.

**Figure 5:**
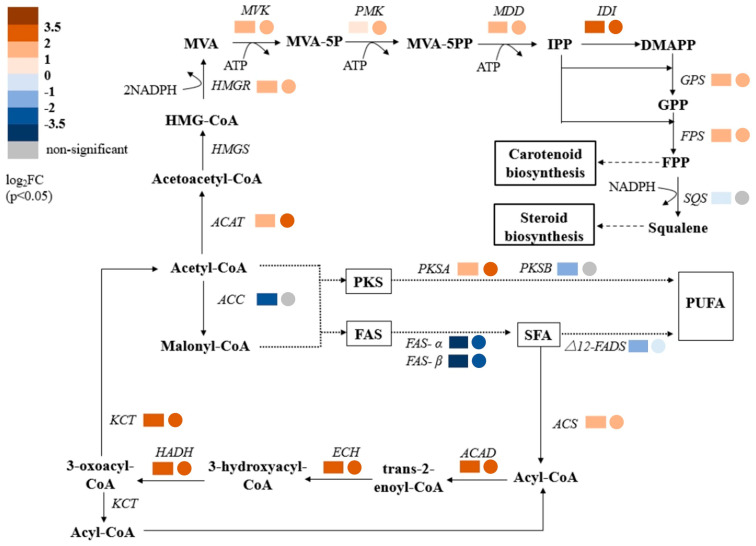
Transcriptomic analysis of the squalene and fatty acid biosynthesis pathways in *Thraustochytrium* sp. ATCC 26185. For each enzyme, the rectangle on the left represents the NS144 vs. LS144 comparison group, while the circle on the right indicates the HS144 vs. LS144 group.

Significant upregulation of MVA pathway genes was observed under both 20 and 45 g/L NaCl (Figure 5). Expression of acetyl-CoA acetyltransferase (ACAT), which catalyzes the initial step in the MVA pathway by condensing two acetyl-CoA molecules into acetoacetyl-CoA, increased by 3.56 and 4.09 fold, respectively. This indicates an enhanced carbon flux toward the MVA pathway under saline stress. Other key enzymes, including 3-hydroxy-3-methylglutaryl-CoA reductase (HMGR), mevalonate kinase (MVK), phosphomevalonate kinase (PMK), and mevalonate diphosphate decarboxylase (MDD), also showed coordinated upregulation, with fold increases ranging from 1.76 to 3.41 (Table 5). The upregulation of HMGR, a well-established rate-limiting enzyme in squalene biosynthesis, is consistent with studies in *Saccharomyces cerevisiae,* where HMGR overexpression significantly enhanced squalene yield [40].

The most pronounced change was in isopentenyl diphosphate isomerase (IDI), whose expression increased by 4.74 and 5.48 fold at 20 and 45 g/L NaCl, respectively. IDI plays a crucial role in balancing the isoprenoid precursors IPP and DMAPP, and its strong induction likely facilitates increased flux toward FPP and squalene biosynthesis [41]. Interestingly, the expression of squalene synthase (SQS), responsible for converting FPP to squalene, was downregulated by 1.58 fold at 20 g/L NaCl and remained unchanged at 45 g/L. Despite this, squalene content increased, suggesting that upstream precursor accumulation and potential repression of downstream squalene-consuming pathways (e.g., sterol biosynthesis) may contribute to net squalene accumulation. This aligns with prior findings, where the downregulation of squalene monooxygenase (*Erg1p*) led to increased squalene retention [42,43].

Concurrently, expression changes in fatty acid biosynthesis pathways revealed a potential reallocation of acetyl-CoA toward squalene production. At 20 g/L NaCl, acetyl-CoA carboxylase (ACC), the rate-limiting enzyme converting acetyl-CoA to malonyl-CoA, was downregulated by 8.99 fold (Table 5), indicating reduced carbon flux toward fatty acid biosynthesis. FAS-α and FAS-β, key enzymes in the fatty acid synthase (FAS) pathway, were also downregulated by 13.96 and 16.65 fold, respectively, suggesting diminished saturated fatty acid (SFA) synthesis. In contrast, PKSA expression decreased by 3.83 fold, while PKSB increased by 2.81 fold, implying a partial shift toward polyunsaturated fatty acid (PUFA) biosynthesis via the PKS pathway (Table 5).

At 45 g/L NaCl, the ACC expression remained unchanged, though FAS-α and FAS-β remained downregulated (6.71 and 8.89 fold, respectively). Meanwhile, PKSB was further upregulated by 3.18 fold, while PKSA expression remained stable (Table 5). These changes are consistent with the increased PUFA content previously observed at this salt concentration. Collectively, these results suggest that high-salt stress induces a redistribution of carbon flux, away from fatty acid synthesis and toward the MVA pathway, favoring squalene accumulation while selectively enhancing PUFA biosynthesis through PKS regulation. However, while these transcriptional and metabolic correlations provide strong indirect evidence, direct fluxomic validation is essential to confirm the proposed carbon reprogramming. To address this, future studies could employ ^13^C flux analysis, which would enable precise quantification of carbon flow between competing pathways under salt stress.

Overall, high-salinity stress orchestrates a complex reprogramming of lipid metabolism in marine oleaginous protists by coordinately regulating squalene and fatty acid biosynthesis. The observed upregulation of upstream MVA pathway genes, including ACAT, HMGR, and IDI, under elevated NaCl concentrations facilitates increased precursor supply for squalene production. Simultaneously, reduced expression of ACC and FAS genes under moderate salt stress suggests a diversion of acetyl-CoA away from fatty acid biosynthesis. This trade-off between fatty acid and isoprenoid pathways has been reported in other systems, where a redirection of carbon flux can enhance isoprenoid production at the expense of fatty acids [44]. A comprehensive study on *Aurantiochytrium* sp. reported key metabolic adjustments under moderate salinity fluctuations, showing that SQS gene upregulation boosted squalene titers under optimal saline conditions [5]. Additionally, selective upregulation of PKSB under high salinity implies a regulatory mechanism promoting PUFA synthesis to maintain membrane fluidity under osmotic stress, as observed in *Dunaliella salina* and other halotolerant species [45]. These findings underscore the metabolic plasticity of *Thraustochytrium* and highlight salinity as a viable environmental lever to boost high-value isoprenoid and PUFA production.

## 3. Materials and Methods

### 3.1. Microorganism and Culture Conditions

*Thraustochytrium* sp. ATCC 26185 was purchased from the American Type Culture Collection (ATCC, Manassas, VA, USA) and maintained on a medium of 30 g/L glucose, 2.5 g/L yeast extract, 2 g/L monosodium glutamate, 25 g/L NaCl, 5 g/L MgSO_4_·7H_2_O, 1 g/L KCl, 0.1 g/L NaHCO_3_, 0.3 g/L CaCl_2_, 0.3 g/L KH_2_PO_4_, 2.9 mg/L FeCl_3_, 0.6 mg/L ZnSO_4_·7H_2_O, 8.6 mg/L MnSO_4_·7H_2_O, 0.26 mg/L CoCl_2_·6H_2_O, and 0.02 mg/L CuSO_4_·5H_2_O. To prepare the seed culture, a single colony from the plate was inoculated into a 100 mL shake flask filled with 50 mL of the fresh medium. The culture was grown for 24 h at 28 °C with 170 rpm. The obtained cells were then transferred to fresh medium at a 1:10 dilution (*v*/*v*) in 1000 mL shake flasks containing 500 mL of medium and incubated for 48 h under the same conditions. Cells were harvested by centrifuging at 4000 rpm for 10 min. The resulting cell pellets were washed twice with fresh medium without NaCl and subsequently transferred into different flasks of 100 mL containing 50 mL medium (0, 20, and 45 g/L NaCl were individually added into each flask). The cells with a cell density of OD_600_ = 0.3 were cultivated for 192 h in a shaker incubator set at 28 °C and 170 rpm to conduct NaCl experiments. Samples of each NaCl concentration were taken every 24 h during the course of cultivation for different biochemical analyses. For each experimental condition, three biological replicates were independently established.

### 3.2. Biochemical Analyses

#### 3.2.1. Determination of Dry Cell Weight

Dry cell weight (DCW) was determined using the gravimetric method described in our previous study [46]. The collected cell cultures were centrifuged at 5000 rpm for 10 min and washed twice with sterile distilled water. The resulting pellets were lyophilized for 48 h at −56 °C and 0.47 mbar using a freeze-drying system (Christ, Osterode am Harz, Germany). The weight of freeze-dried cells was measured with an electronic balance, and then, they were stored at −80 °C for future use. The DCW was calculated by measuring the weight difference before and after drying.

#### 3.2.2. Determination of Glucose Concentration

The residual glucose concentration of the fermentation broth was determined using the DNS method [47]. In brief, 0.5 mL of diluted centrifugation supernatant obtained from fermentation liquid was transferred into a 15 mL centrifuge tube, followed by adding 1.5 mL DNS solution. The centrifuge tubes were incubated in a boiling water bath at 95 °C for 5 min and afterward cooled down rapidly with running water to room temperature. Then, 10 mL ultra-pure water was added into the tubes, and the mixtures were thoroughly mixed on a vortex mixer. Finally, the OD value was measured by a visible spectrophotometer (Jinghua, Shanghai, China) at 540 nm, and the glucose concentration was calculated according to the standard curve.

#### 3.2.3. Squalene Analysis

Quantifying squalene was performed as per the method described in our previous study [14]. Cells (5 mL of culture from independent 50 mL flasks) collected at each time point were transferred into 15 mL centrifuge tubes and then centrifuged at 5000 rpm for 10 min. The cell pellets were washed twice with distilled water. To extract squalene, 2 mL of chloroform–methanol (1:2 *v*/*v*) was mixed with the pellets obtained from the previous step, and the resulting mixture was vortexed for 3 min. Subsequently, 2 mL acetonitrile and 4 mL hexane were added to the above mixture, followed by further vortexing for 1 min and centrifuging at 4000 rpm for 2 min. The squalene extracted into the hexane supernatant phase was transferred into a new tube and adjusted to a specific volume by using hexane. For squalene quantification, 0.5 mL of the upper hexane layer was injected into the Agilent 7890B gas chromatography system (Agilent, Santa Clara, CA, USA) equipped with HP-5MS UI column (30 m ×250 μm × 0.25 μm) and a flame ionization detector with nitrogen as a carrier gas. The temperature of the GC oven was set to 90 °C for 1 min, then increased to 300 °C at a rate of 25 °C/min and maintained at 300 °C for 7 min. The chromatographic data were collected and processed using Agilent OpenLAB CDS ChemStation Edition A.02.05.021 software. The squalene concentrations were calculated based on the squalene standard curve by using the external standard method.

#### 3.2.4. Fatty Acids Analysis

Fatty acid methyl esters (FAME) were prepared by using the method described previously [48]. To extract lipids, 100 μL of 1 mg/mL nonadecanoic acid (C19:0, dissolved in hexane) were added as an internal standard to the lyophilized cells (50~100 mg, from Section 3.2.1) and mixed with 2 mL of 4% sulfuric acid in methanol (*v*/*v*). The resulting mixtures were vortexed for 1 min and incubated in a water bath at 80 °C for 1 h for transesterification. After the samples were cooled to room temperature, 1 mL ultrapure water and 1 mL hexane were added. The solvent mixture was vortexed for 1 min and centrifuged at 4000 rpm for 2 min. For analysis, 0.5 mL of the upper hexane layer containing FAME were injected into the Agilent 7890B gas chromatography system [49]. The GC was equipped with a DB-WAX column (60 m × 320 μm × 0.15 μm) and a flame ionization detector. The column temperature was held at 50 °C for 1 min, followed by a temperature increase at a rate of 25 °C/min to 175 °C, then 3 °C/min to 220 °C, kept for 5 min, and finally increased temperature at the rate of 2 °C/min to 230 °C and maintained for 11 min. In general, the area of a sample peak determined by GC is directly proportional to the content of the corresponding component. In our experiment, the amount of the internal standard (C19:0) added was known. Thus, based on the ratio of peak areas, the relative proportions of each component were calculated, allowing for the determination of both the total fatty acid content and the quantities of individual components.

### 3.3. Transcriptomics Analysis

To conduct the transcriptomics analysis, cell cultures under three NaCl concentrations were collected at 72 h and 144 h. Three biological replicates were performed for each treatment condition. Each sample was obtained by taking 10 mL from the culture for subsequent processing. The cultures were centrifuged at 5000 rpm and 4 °C for 1 min. The cell pellets were then washed once with PBS buffer (pre-cooled to 4 °C). The resulting pellets were quickly frozen in liquid nitrogen for 1 h, then stored in a −80 °C freezer. For further analysis, a part of the samples was used for RNA extraction, and the remaining part was saved for metabolomics analysis.

The frozen cells were sent to Hangzhou Lianchuan Biotechnology Co., Ltd., for transcriptomic analysis. Briefly speaking, total RNA extraction for each sample was performed using Trizol reagent (Invitrogen, Carlsbad, CA, USA) following the manufacturer’s instructions. The quantity and purity of the total RNA were assessed using the Bioanalyzer 2100 and the RNA 1000 Nano LabChip Kit (Agilent, San Jose, CA, USA), with a RIN number >7.0. Five micrograms of total RNA were used to purify poly(A) mRNA using RNA poly-T oligo-attached magnetic beads. After two rounds of purification, the mRNA was fragmented into small pieces using divalent cations at elevated temperatures. The RNA fragments were then reverse-transcribed to form the final cDNA library according to the mRNASeq sample preparation kit (Illumina, San Diego, CA, USA), with an average insertion length of 300 ± 50 bp. Paired-end sequencing was performed on an Illumina Novaseq™ 6000 (LC Sciences, Houston, TX, USA).

After obtaining the raw sequencing data, it is important to carry out pre-processing steps to ensure the quality and reliability of the data. Initially, reads containing adaptor contamination, low quality, or undetermined bases were removed using Cutadapt and in-house Perl scripts [50]. The quality of the sequences, including Q20, Q30, and GC-content, was verified using FastQC (http://www.bioinformatics.babraham.ac.uk/projects/fastqc/, accessed on 19 August 2022). Only high-quality, clean data were used for downstream analyses. De novo assembly of the transcriptome was performed using Trinity 2.4 [51]. Transcripts were clustered based on shared sequence content, and the longest transcript in each cluster was selected as the ‘gene’ sequence (also known as Unigene). All assembled Unigenes were aligned against the non-redundant (Nr) protein database, Gene Ontology (GO), SwissProt, Kyoto Encyclopedia of Genes and Genomes (KEGG), and eggNOG databases using DIAMOND, with an E-value threshold of <10^−5^ [52]. After the assembly and annotation of the transcriptome, the next step is to quantify the gene expression levels and identify the differentially expressed genes. Gene expression levels were normalized by calculating the TPM [53] using Salmo [54]. Differentially expressed genes (DEGs) were identified with a *p*-value < 0.05 and an absolute log2 (fold change) > 1 using the R package edge (Version 1.30.1) [55].

### 3.4. Metabolomics Analysis

The time points and methods for collecting cells and naming the samples were consistent with those described in the transcriptome analysis. The frozen cells obtained from the previous section were sent to Hangzhou Lianchuan Biotechnology Co., Ltd. (Hangzhou, China), for non-targeted metabolomics analysis. For the sample preparation, the cell pellets were thawed on ice, and metabolites were extracted with a 50% methanol buffer. Briefly, 12 μL of precooled 50% methanol was added to 20 μL of the pellets, vortexed for 1 min, and incubated at room temperature for 10 min to precipitate the proteins. The mixture was then stored at −20 °C overnight. Afterward, the extraction mixture was centrifuged at 4000× *g* for 20 min, and the supernatant was transferred into 96-well plates. Quality control (QC) samples were prepared by pooling equal volumes of individual samples (10 μL), and these samples were stored at −80 °C before the LC-MS analysis. The extracted samples were randomly arranged. Non-targeted metabolomic profiling of the samples was performed using a high-resolution mass spectrometer. QC samples were inserted at the beginning, middle, and end of the sample sequence to assess the technical reproducibility of the experiment.

Metabolites were detected using liquid chromatography–mass spectrometry (LC-MS) [56]. First, a Vanquish Flex UHPLC system (Thermo Fisher, Bremen, Germany) equipped with an ACQUITY UPLC T3 column (100 mm × 2.1 mm × 1.8 μm) was used for reversed-phase chromatographic separation. The column oven was maintained at 35 °C, and the mobile phase flowed at 0.4 mL/min, consisting of solvent A (water with 0.1% formic acid) and solvent B (acetonitrile with 0.1% formic acid). The gradient elution conditions were as follows: 5% B from 0 to 0.5 min; 5% to 100% B from 0.5 to 7 min; 100% B from 7 to 8 min; 100% to 5% B from 8 to 8.1 min; and 5% B from 8.1 to 10 min. A Q-Exactive high-resolution tandem mass spectrometer (Thermo Fisher, Bremen, Germany) was used to detect the metabolites eluted from the column in both positive and negative ion modes. Precursor spectra were collected in the 70–1050 *m*/*z* range at 70,000 resolution with an AGC target of 3 × 10^6^ and a maximum injection time of 100 ms. Fragment spectra were collected at 17,500 resolution with an AGC target of 1 × 10^5^ and a maximum injection time of 80 ms [57].

Finally, a series of specialized software tools was used to conduct data analysis. Metabolome data analysis was performed by Biomarker Technologies (Beijing, China). MSConvert software (Version 3.0.10385) converted the original MS data into a readable mzXML format [58]. XCMS software (Version 1.24.1) was used for peak extraction and quality control. Extracted substances were annotated using CAMERA and identified by metaX software (Version 2.68) [59]. Primary mass spectrum information was used for identification, and secondary mass spectrum information was matched with an in-house standards database. Candidate substances were annotated using HMDB, KEGG, and other databases. Additionally, metaX software was used to quantify and screen differential metabolites. Metabolites with a VIP (variable importance for projection) score ≥ 1, a *p*-value from the *t*-test < 0.05, and a fold change (FC) ≥ 2 or FC ≤ 1/2 were considered differential metabolites.

### 3.5. Statistical Analysis

All experiments were performed with three independent biological replicates. Data are presented as the mean ± standard deviation (SD). Statistical significance between the control and treatment groups was determined using an unpaired, two-tailed Student’s t-test. A *p*-value of less than 0.05 was considered statistically significant. Different asterisks indicate the level of significance: * *p* < 0.05, ** *p* < 0.01, and *** *p* < 0.001.

## 4. Conclusions

This study elucidates the salinity-dependent metabolic responses of *Thraustochytrium* sp. ATCC 26185. Moderate salinity (20 g/L NaCl) optimized biomass production and glucose utilization, indicating enhanced cellular activity. In contrast, squalene accumulation increased with salinity, peaking at 45 g/L NaCl, suggesting a regulatory role of NaCl in isoprenoid biosynthesis. Integrative transcriptomic and metabolomic analyses revealed that high salinity activates central carbon metabolism, substrate-level phosphorylation, and oxidative phosphorylation, supporting ATP generation to meet the energetic demands of osmotic stress adaptation—consistent with responses observed in other halotolerant microbes. Concurrently, fatty acid metabolism was reprogrammed: saturated fatty acid (SFA) synthesis was downregulated, while polyunsaturated fatty acid (PUFA) biosynthesis was enhanced, reallocating carbon flux to improve salt tolerance. Notably, integrated transcriptomic and metabolomic analyses in this work provide further molecular evidence that high NaCl concentrations stimulate squalene accumulation by upregulating the mevalonate (MVA) pathway. Key enzymes, including ACAT, HMGR, and IDI, were significantly induced under salt stress, increasing the carbon flux toward squalene biosynthesis. These findings align with prior reports that ATP availability constrains terpene biosynthesis in ATCC 26185 and that salt-induced energy metabolism may alleviate this limitation.

## Figures and Tables

**Table 1:** Statistics of transcriptome sequencing data quality control results.

Sample	Raw_Reads	Raw_Bases	Valid_Reads	Valid_Bases	Valid%	Q30%	GC%
LS72	41,205,283	6.18	38,987,255	5.43	94.61	93.86	63.61
NS72	40,762,481	6.11	39,130,041	5.45	96.00	93.89	63.89
HS72	40,343,527	6.05	38,487,931	5.36	95.39	93.74	63.92
LS144	41,151,169	6.17	39,584,795	5.51	96.19	93.83	63.64
NS144	43,231,437	6.48	41,160,139	5.73	95.21	93.24	63.88
HS144	42,497,021	6.37	40,259,607	5.61	94.73	93.16	63.91

**Table 2:** Overview of the trinity assembly results of *Thraustochytrium* sp. ATCC 26185.

All	GC%	Min Length	Median Length	Max Length	Total Assembled Bases	N_50_
27,812	62.65	201	641	40,915	33,143,887	2110

**Table 3:** Differentially expressed genes between different comparison groups of *Thraustochytrium* sp. ATCC 26185.

Sample	Total DEGs	Upregulated DEGs	Downregulated DEGs
NS72_vs_LS72	2649	877	1772
HS72_vs_LS72	2935	1358	1577
HS72_vs_NS72	1312	891	421
NS144_vs_LS144	4012	1835	2177
HS144_vs_LS144	4542	1666	2876
HS144_vs_NS144	1407	221	1186

**Table 4:** Results of metabolic ion identification.

Mode	All	MS2	HMDB	KEGG	Annotated
negative	7907	403	3640	2921	4321
positive	14,410	716	6659	4887	7635

Mode: The detection mode of the mass spectrometry instrument for substances. All: The total number of identified metabolic ions; MS2: The number of metabolic ions annotated to secondary metabolites; HMDB: The number of primary metabolites annotated to the HMDB database; KEGG: The number of primary metabolites annotated to the KEGG database; Annotated: The number of annotated primary metabolites.

**Table 5:** Statistical table of key enzyme gene expressions in the squalene synthesis pathway and the fatty acid synthesis pathway at different concentrations of NaCl by *Thraustochytrium* sp. ATCC 26185.

Enzyme	Gene Expression Quantity (TPM)			NS144-vs-LS144			HS144-vs-LS144		
	LS144	NS144	HS144	FC	log_2_FC	*p* Value	FC	log_2_FC	*p* Value
ACAT	199.26	709.89	814.95	3.56	1.91	7.70 × 10^−21^	4.09	2.20	1.54 × 10^−26^
HMGR	71.3	178.93	188.62	2.51	1.44	4.02 × 10^−12^	2.65	1.59	9.02 × 10^−11^
MVK	79.79	219.75	272.24	2.75	1.57	3.25 × 10^−12^	3.41	1.96	6.34 × 10^−20^
PMK	18.67	32.82	44.91	1.76	0.93	8.81 × 10^−5^	2.41	1.47	2.19 × 10^−15^
MDD	36.22	70.62	83.5	1.95	1.08	5.61 × 10^−6^	2.31	1.40	1.00 × 10^−8^
IDI	59.66	282.93	326.72	4.74	2.26	3.43 × 10^−28^	5.48	2.55	1.80 × 10^−36^
GPS	126.33	252.00	348.26	1.99	1.11	1.93 × 10^−5^	2.76	1.66	2.45 × 10^−11^
FPS	126.33	252.00	348.26	1.99	1.11	1.93 × 10^−5^	2.76	1.66	2.45 × 10^−11^
SQS	118.77	75.25	86.25	−1.58	−0.54	0.017	-	-	0.36
ACC	208.67	23.21	80.13	−8.99	−3.05	2.58 × 10^−12^	-	-	0.16
FAS-α	214.39	15.36	31.96	−13.96	−3.69	8.33 × 10^−11^	−6.71	−2.58	0.0037
FAS-β	90.91	5.46	10.23	−16.65	−3.92	2.91 × 10^−11^	−8.89	−2.96	0.0003
PKSA	188.28	49.16	149.78	−3.83	1.79	0.00011	-	-	0.85
PKSB	93.23	262.3	296.58	2.81	1.60	1.61 × 10^−7^	3.18	1.86	1.39 × 10^−18^
Δ12 desaturase	539.44	239.15	281.48	−2.26	−1.08		−1.92	−0.75	0.0061

## Data Availability

All data supporting this study are available within the paper. For additional raw data files, they can be obtained from the corresponding author upon reasonable request.
